# Identifying Silver Linings During the Pandemic Through Natural Language Processing

**DOI:** 10.3389/fpsyg.2021.712111

**Published:** 2021-09-03

**Authors:** Juan Antonio Lossio-Ventura, Angela Yuson Lee, Jeffrey T. Hancock, Natalia Linos, Eleni Linos

**Affiliations:** ^1^Department of Dermatology, Stanford University, Stanford, CA, United States; ^2^Machine Learning Team, National Institute of Mental Health, National Institutes of Health, Bethesda, MD, United States; ^3^Department of Communication, Stanford University, Stanford, CA, United States; ^4^FXB Center for Health and Human Rights, Harvard University, Cambridge, MA, United States

**Keywords:** silver linings, protective factors, COVID-19, natural language processing, topic modeling, sentiment analysis

## Abstract

COVID-19 has presented an unprecedented challenge to human welfare. Indeed, we have witnessed people experiencing a rise of depression, acute stress disorder, and worsening levels of subclinical psychological distress. Finding ways to support individuals' mental health has been particularly difficult during this pandemic. An opportunity for intervention to protect individuals' health & well-being is to identify the existing sources of consolation and hope that have helped people persevere through the early days of the pandemic. In this paper, we identified positive aspects, or “silver linings,” that people experienced during the COVID-19 crisis using computational natural language processing methods and qualitative thematic content analysis. These silver linings revealed sources of strength that included finding a sense of community, closeness, gratitude, and a belief that the pandemic may spur positive social change. People's abilities to engage in benefit-finding and leverage protective factors can be bolstered and reinforced by public health policy to improve society's resilience to the distress of this pandemic and potential future health crises.

## Introduction

The COVID-19 pandemic has presented an unprecedented challenge to human welfare. Despite rising numbers of vaccinations, cases continue to surge in many parts of the world causing both our healthcare systems and our collective well-being to be stretched to their limits (Nelson et al., 2020). In the face of this tragedy, individuals have had to confront profound grief, loss, and uncertainty about the future that can severely impact their mental health (Mumphrey and Kelleher, 2020).

The challenges of coping with a global pandemic can be seen in the rise of depression, acute stress disorder, anxiety disorders, and worsening levels of subclinical psychological distress, all of which have substantially increased in prevalence since the pandemic's onset (Brooks et al., 2020; Burhamah et al., 2020; Czeisler et al., 2020; Huang and Zhao, 2020). In response, public health organizations like the World Health Organization have highlighted the need to provide psychosocial support to individuals struggling with their mental health during and in the aftermath of the pandemic (World Health Organization, 2020).

Finding ways to support individuals' mental health may be particularly difficult during a pandemic, when many conventional methods of support such as peer-to-peer interaction, social belonging, and community activities may no longer be safe or accessible (Campion et al., 2020). Furthermore, coping mechanisms such as short-term problem-solving or emotion management may be insufficient for meeting the needs of the moment. As a result, solutions are needed that can mitigate threats to mental health by building on existing protective factors and encouraging positive coping strategies, without requiring activities that may run counter to COVID-19 prevention measures.

One opportunity for intervention to protect individuals' health and well-being is to identify the existing sources of strength and hope that have helped people persevere through the pandemic, so that public health policy and messaging can fortify these strengths. Colloquially known as “silver linings,” the act of finding positive ways that one's life has changed as a result of a traumatic or stressful event is known as *benefit-finding* in the study of resilience (Folkman and Moskowitz, 2007). This is a particular form of meaning-focused coping that helps people process difficult situations by changing how they make sense of the situation in their own lives or in the world around them (Folkman, 2008).

The protective effects of benefit-finding have largely been found in the context of bereavement after losing loved ones, anxiety about health threats, and surviving natural disasters—all of which are reflected in the COVID-19 pandemic. Indeed, early research on resilience in the pandemic has found that benefit-finding is associated with reductions in psychological distress. A recent study by Cox et al. found that increased benefit-finding was associated with not only greater life satisfaction, meaning, and vitality but also decreased reports of depression and stress (Cox et al., 2021).

In light of calls to understand the role of positive coping strategies in the pandemic and in the path forward as the world heals (Burke and Arslan, 2020), we sought to identify and characterize the types of benefits found by individuals during the COVID-19 pandemic. We asked:

**RQ1:** What, if any, are the benefits, or ‘silver linings', that have helped people persevere through the COVID-19 pandemic?

Based on previous work on protective factors linked to increased resilience during COVID-19, we predicted that two factors that would emerge in our analysis would be time spent with loved ones and gratitude.

In this paper, we use a mixed-method approach that includes computational natural language processing (NLP) methods, including sentiment analysis and topic modeling, along with qualitative thematic content analysis to identify these protective factors from a survey. NLP methods have long been used to explore people's thoughts and feelings during emotional events and crises by processing patterns in language use at scale—an affordance that may be particularly valuable during rapidly evolving situations such as the COVID-19 pandemic (Cohn et al., 2004; Tausczik et al., 2012; Wolohan, 2020). Similarly, in-depth thematic content analysis is often used to identify more granular patterns in text responses.

As it becomes increasingly clear that addressing the full impact of the pandemic requires implementation of public health policy on mental health (Bearden and Karlsgodt, 2021), the goals of this paper are 2-fold. We aim to identify these silver linings to understand how people find solace in times of crisis, and to consider how we may carry forward positive societal changes caused by the pandemic into a post-COVID world. Understanding the sources of hope that have helped people weather the psychological toll of COVID-19 can inform public health policy to improve society's resilience to the distress of this pandemic and future health crises.

## Methods

### Procedure

In March 2020, we launched an online survey on three social media platforms (Twitter, Facebook, and Nextdoor) to identify the impact of the COVID-19 pandemic on participants' lives. Any individual 18 and older was eligible to participate. They could participate by clicking on the link to the survey embedded in the social media post upon seeing the recruitment materials. The survey was approved by Stanford's Institutional Review Board and all participants consented to the study.

The survey included a total of 21 questions including demographics and the impact of COVID-19 on individuals' lives, which is reported in Nelson et al. (2020). In this work, we focused on the evaluation of free-text responses to the question “Although this is a challenging time, can you tell us about any positive effects or 'silver linings' you have experienced during this crisis?” The authors agreed to make the data available upon reasonable request.

### Participants

We recruited a convenience sample of 4,582 participants over the 7-month period from March to September 2020. We excluded 1,469 participants who did not respond to the silver-linings question that was central to our analysis, resulting in a final sample size of 3,113 responses. The average age of participants was 47.41 ± 15.2 years (range: 18–99), 71.5% were women, 59% were white as shown in Table 1. Participants of 68 different countries completed the survey, mostly from the U.S. (82.8%) followed by Guatemala (2.8%), Canada (2%), New Zealand (1.7%), and the U.K. (1.4%).

**Table 1:** Participant demographics and sentiment analysis results (18 < = age < = 99).

**Characteristic**	**ALL responses to silver lining** ** (*N* = 3,113)**	**polarity** **Score** **0.22**	**Relevant topics**
Gender			
Female	71.5%	0.23	Family time, sense of community, quality time with spouse
Male	25.4%	0.19	Time with family, time with wife, work from home
Others^‡^	3.1%	0.22	Anxiety stress of traffic, art therapy, alcohol to-go
Age, years, (mean ± SD)	47.41 ± 15.2		
18–30	14.7%	0.23	Work from home, family over zoom, air pollution from car
31–45	31.3%	0.24	Time with family, work from home, money on gas, virtual happy hours
46–64	38.4%	0.21	Spending time with family, cooking at home, college kids
65+	12.3%	0.18	Family members, contact with family, intense appreciation for health care
Other	3.3%	0.22	Close look at family budgeting, time at home, close look at family
Race			
White	59.0%	0.22	Marked improvement in air quality, sense of community, contact with family
Asian^*^	6.4%	0.25	Daily walks, zero commute time, appreciation for family, action plan for career
Black	5.7%	0.23	Covid-19 like HIV, annual income over $80k, college kids, basic clothing
Latin/Hispanic	1.3%	0.24	Bay area traffic, quality time with family, video chats
Other^†^	27.6%	0.19	Trabajar desde mi casa, ganar dinero diario debe ser un derecho
Countries			
U.S.	82.8%	0.22	Time with family, money on gas, time for exercise, productive working from home
Guatemala	2.8%	0.21	Trabajar desde mi casa, family time, ganar dinero diario
Canada	2.0%	0.19	Family at home, public health, cooking at home, favorite Canadian, tea household chores
New Zealand	1.7%	0.22	Great sense of community concern, flexible sleep work schedule, good government leadership
U.K.	1.4%	0.20	Lockdown light in UK, cook from scratch, appreciation of healthcare
Month			
March	53.8%	0.23	Family budgeting, appreciation for job, family over zoom
April	37.7%	0.22	Clean air, time for exercise, time for reading, cooking at home, family time
May	5.4%	0.15	Family time, yard work, break from pollution, proper management of money
June	1.0%	0.26	Acrylic painting, anxious temperament, watch Netflix
July	1.0%	0.25	High school, family members, home projects
August	0.8%	0.12	Video chat, behavior around handwashing, local air quality
September	0.3%	0.25	Performative nature of office work, different coffee subscriptions

* *Asian includes Asian and Pacific Islander;*

† *Other race category includes Other, Native American, and those with empty values.*

‡ *Other gender category includes other and those with empty values*.

### Statistical Analyses

We used a combination of computational and qualitative methods of natural language processing to identify the themes in participants' text responses. Given the large size and the richness of the dataset, our approach sought to leverage the complementary strengths of both methods. Computational methods of natural language processing were used to automatically evaluate and extract themes from the responses at scale, and to examine the structure and distribution of silver linings themes across responses. Qualitative methods of thematic content analysis were used to confirm the themes, to identify more fine-grained, specific topics, and to select exemplar quotes for each theme.

#### Computational Analyses

**Figure 1:**
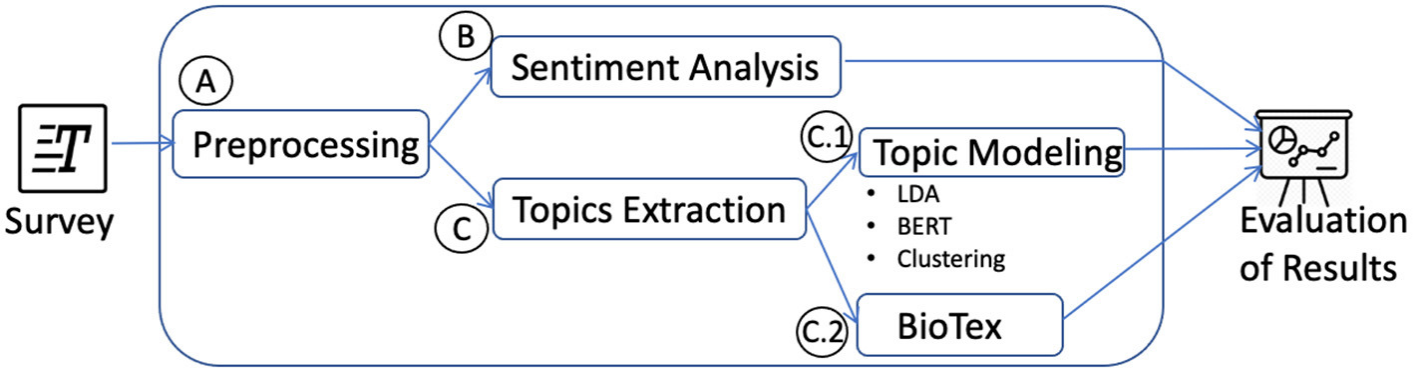
Workflow of the Natural Language Processing methods applied to analyze the text of the survey.

This section describes the computational methods of natural language processing used to automatically evaluate the text of the silver linings responses from the online survey. As seen in Figure 1, we outlined the basic steps of: (A) Preprocessing; (B) Sentiment Analysis; and (C) Topics Extraction.

**(A) Preprocessing**:

We preprocessed the dataset of survey responses to preserve the confidentiality and integrity of protected health information (PHI). Thus, we applied the following rules of preprocessing: (1) specific occurrences for email addresses were replaced with generic tokens; (2) named entities such as people's names were also replaced with generic tokens; (3) text was lemmatized; (4) all text was changed to lowercase; (5) stop-words were suppressed, such as “*an*,” “*also*,” etc.; and (6) links were omitted.

**(B) Sentiment Analysis:**

The aim of our analysis is to measure the intensity of responses related to silver lining topics in the early stages of the pandemic. Thus, we sought to understand the emotional properties of participants' responses with sentiment analysis. The objective of sentiment analysis is to discover emotion, opinion, and subjectivity expressed in a piece of text (Feldman, 2013). In this study, we used TextBlob, a Python-based library for processing textual data, to explore patterns in how people discussed silver linings in the pandemic (Loria, 2018). TextBlob provides an API for tasks such as part-of-speech tagging, lemmatization, sentiment analysis, among others. For sentiment analysis, TextBlob returns a score called polarity within the range [−1, 1]. Each response is evaluated in terms of polarity where −1 represents very negative and +1 represents very positive.

**(C) Topic Extraction:**

In order to identify the types of silver linings participants reported experiencing, we used NLP methods of topic modeling and clustering to digest, analyze, and automatically extract topics from survey responses. Topic modeling aims to discover latent topics inherent to a set of textual responses, by identifying groups of words that frequently occur together (Lossio-Ventura et al., 2021). Clustering methods group texts based on their similarity.

In this study, we characterized the responses via feature extraction by employing a combination of the latent Dirichlet allocation model (LDA) and semantic representation transformers called Bidirectional Encoder Representations from Transformers (BERT), then grouped similar responses using k-means clustering. Based on studies demonstrating that this combination improved performance on numerous language analysis tasks (Rangrej et al., 2011; Peinelt et al., 2020; Xie et al., 2020), this approach allowed us to better reveal the most common types of silver linings in participants' responses.

The LDA is a probabilistic model that describes a set of texts as a mixture of distinct topics, which are represented as a mixture of distributions of words (Blei et al., 2003). LDA uses a Bayesian approach to learn these distributions, which allows it to compute the probability that a text belongs to a given topic. We implemented this with Gensim version 4.0.0, an open-source library for unsupervised topic modeling (Rehurek and Sojka, 2010). Parameters of LDA were set as suggested in previous studies to obtain optimal performance on short texts. The hyper-parameters of LDA were set to α = 0.05 and β = 0.01. We set the number of iterations to 1,000 and number of topics to 25. Thus, the output vector of each response had a dimension of 25. This produced a vector of probabilities for each participant's survey response, where the elements are probability scores of that response belonging to a particular topic. The sum of all elements of the vector is 1.

BERT is a language representation model released by Google. Recent studies have successfully employed BERT for topic analysis (Zhou et al., 2019; Liu et al., 2020, 2021). BERT enhances the semantic representation of texts with its feature extraction and fine-tuning transfer learning capabilities (Vaswani et al., 2017; Devlin et al., 2019). In this analysis, we used the BERT implementation provided by Hugging Face PyTorch. BERT parameters were set as suggested in Hugging Face PyTorch. Pad was set to the longest sequence in the batch (i.e., 512) and the pretrained model chosen was “bert-base-uncased.” Thus, we encoded each response and obtained a vector embedding of dimension 768.

Next, we entered the LDA and BERT vectors for each survey response into the clustering algorithm. We used one of the most well-known algorithms, k-means (with k-means++ initialization) implemented from Sklearn (MacQueen, 1967). We varied k (i.e., number of clusters) ranging from 2 to 20, and the initialization was set to k-means++. Note that the clustering algorithm received a vector of dimension 793 (25+768). The output of the clustering task is clusters (topics) of responses. To evaluate the performance of the clustering method, we applied a cluster validity index, the Silhouette Coefficient (SC) (Rousseeuw, 1987). The SC index evaluates the clusters/topics based on two aspects: the similarity of responses within the same cluster (cohesion), and the difference between the responses of different clusters. Then we created wordclouds of the most important keywords from each topic. Note that the extracted keywords that represent each topic are single-word terms.

Finally, we used BioTex, an information retrieval tool that extracts multi-word terms (multi-word keywords) from text responses for different languages such as English and Spanish (Lossio-Ventura et al., 2016). BioTex implements several measures to extract multi-word terms, such as TF-IDF, LIDF-value, among others. This tool is based on several linguistic patterns also known as lexical categories such as nouns, adjectives, etc. For instance, BioTex is able to extract multi-word terms such as “*quality time with spouse*” which is composed of the following lexical categories: “*adjective-noun-preposition-noun*.”

#### Qualitative Analyses

To further identify more granular themes from the corpus of open-ended responses in the dataset, we used qualitative methods of thematic content analysis. The coding process was conducted by two trained human coders following Braun and Clarke's (2006) guidance for completing thematic content analysis. First, two members of the research team (AL and EL) familiarized themselves with the data by reading over all of the responses. Next, they met regularly to generate codes and identify initial themes that emerged from a process of discussion and iterative review. These themes were subjected to further review by the research team, yielding a final set of nine themes of silver linings that participants reported as a result of the COVID-19 pandemic. Themes were named, defined, and finalized in a codebook. The manual coding of responses was conducted by two raters: AL who coded 86% of the responses and a research assistant who coded 25% of the responses. Both coders used the same codebook and demonstrated good inter-rater reliability as evaluated by Cohen's kappa (=0.70 −0.94). Upon completion of the coding process, the research team integrated the themes identified into existing literature and identified exemplar quotes.

## Results

### Computational Analyses: Sentiment Analysis and Silver Linings Identified Through Natural Language Processing

The results of the sentiment analysis are presented in Table 1, including comparisons by demographics such as participants' gender, age group, and country of residence, and keyword examples of relevant topics associated with each group. We report the polarity analysis, which is the sentiment scores of mean responses categorized as positive, negative, or neutral in emotion. As expected, we found that the overall sentiment of participants' responses was positive when describing the silver linings they found in the pandemic, with the average polarity score being 0.22. Moreover, in general, women (polarity = 0.23) tend to slightly be more positive than men (polarity = 0.21) and they usually reacted to topics related to kids, followed by parents and partners.

**Figure 2:**
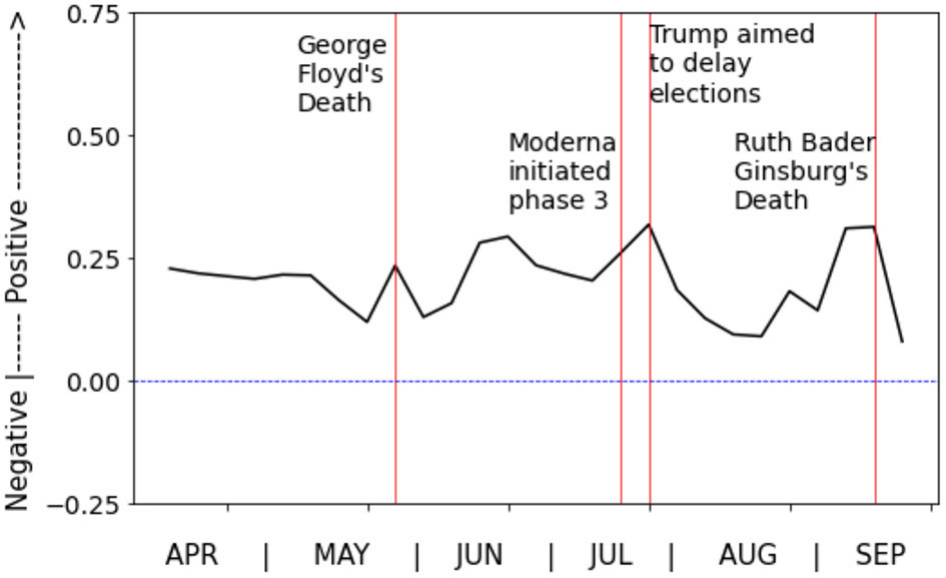
Weekly average of sentiments in responses over time. A sentiment score of −1 represents very negative and +1 very positive.

The weekly average sentiment score of all responses over time are shown in Figure 2. Despite the stressors and tragedies of 2020, the average sentiment appears to remain positive overall. However, we can see that emotions in responses were less positive in the aftermath of distressing events, such as the murder of George Floyd and the death of Ruth Bader Ginsburg. On the other side, emotions in responses were more positive following hopeful events such as the announcement of the creation and testing phases of COVID-19 vaccines.

**Figure 3:**
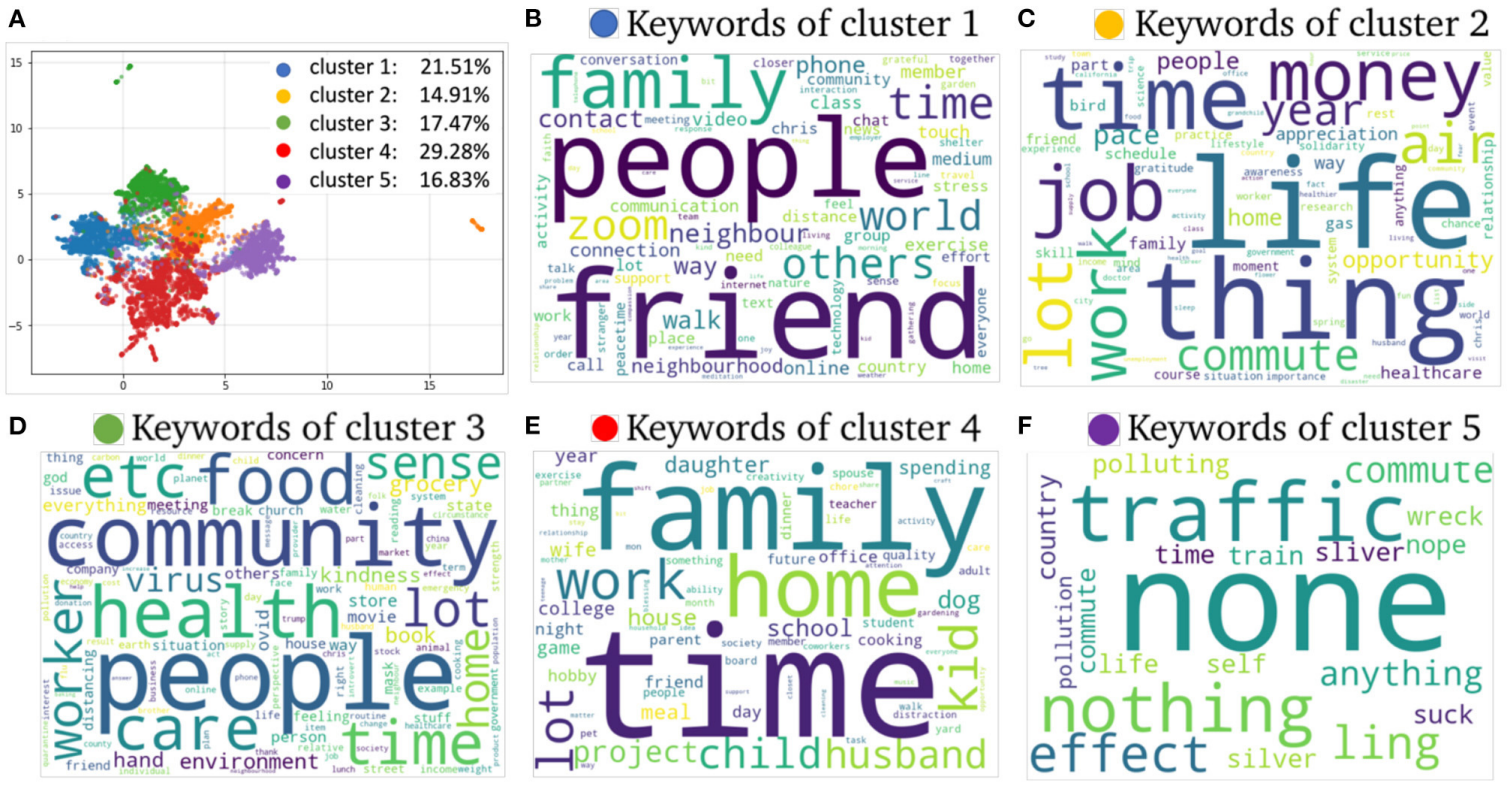
Clustering and most frequent words from free-text responses based on the combination of LDA and BERT embeddings methods. **(A)** Shows the clustering considering 5 clusters (groups) as main topics. Each cluster shows the percentage of number of responses in that group, for instance, cluster 5 contains 16.83% of the total of responses, and **(F)** presents keywords from cluster 5 associated with positive environmental impact, benefits of working from home.

We then identified the types of silver linings people experienced using topic modeling and clustering. Note that we performed experiments using the Silhouette Coefficient (SC) index to measure the performance of the topic extraction step with the number of clusters/topic ranging from 2 to 20 (“k”). The SC obtained the best results when considering 5 clusters; this means the methods automatically created 5 groups where each group contained messages of a topic. Figure 3 depicts the results of clustering (i.e., grouping similar responses) and topic extraction (most frequent words from each topic) of free-text responses based on the combination of LDA and BERT embeddings methods.

**Figure 4:**
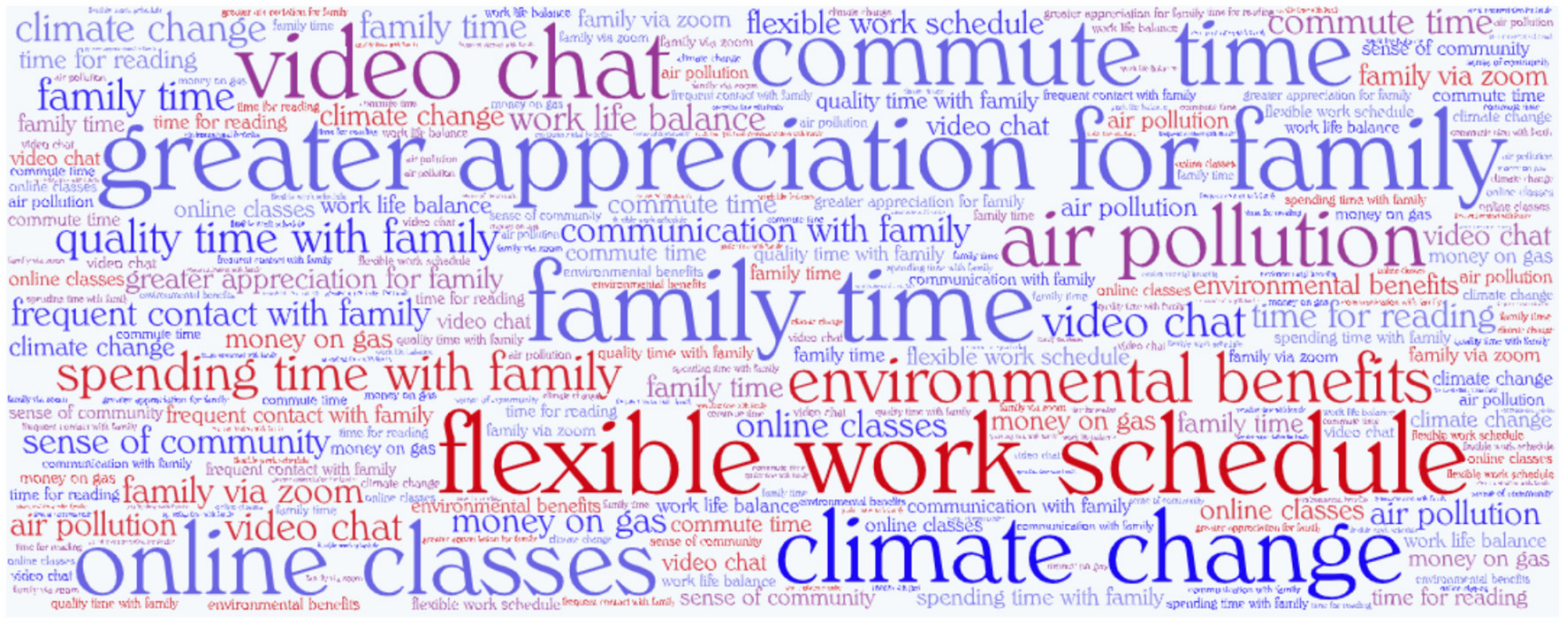
Wordcloud of the most relevant terms (multi-word terms) extracted with BioTex from silver lining responses during the COVID-19 crisis between March and September 2020.

Figure 3A shows the clustering with 5 clusters as main topics. Each cluster shows the percentage of number of responses in that group, for instance, cluster 5 contains 16.83% of the total of responses. Figures 3B–F presents the most frequent words (single-word terms only) from all clusters, for instance, Figure 3F suggests that the cluster is associated with positive environmental impact. Topic modeling shows the different topics of discussion on silver linings are diverse, but the five most common topics that were automatically identified were finding benefits in (1) time with family, (2) work from home and flexible schedules, (3) sense of community and neighborhood, (4) reduced human impact on the environment, and (5) free time to develop skills. We confirmed these topics with BioTex. Figure 4 shows the most relevant terms (multi-word) automatically extracted with BioTex from all response, which are representative from the 5 topics previously identified.

### Qualitative Analyses: Silver Linings Identified Through Thematic Content Analysis

Thematic content analysis allowed us to identify more fine-grained types of silver linings than those extracted with NLP methods. Indeed, the content analysis confirmed the five topics identified through NLP and yielded four additional themes, finding that people reported finding silver linings in (1) feeling gratitude for what they have, (2) improved health literacy (i.e., around correct illness prevention), (3) viewing the pandemic as an impetus for positive social change, and (4) appreciating the communication opportunities made possible by technology.

Table 2 shows the results from the thematic content analysis. Paralleling findings from the NLP analysis, we observe that almost half of participants (46.4%) said that they found benefits in having increased quality time with their loved ones due to staying home during the pandemic. For some, this was driven by changes in their household, as a result of children moving home to their parents or individuals moving in with their partners. For others, this was facilitated by an increased use of technologies like social media and video-chats, which enabled people to connect with their friends and family despite being physically distant. In fact, some people (13.0%) explicitly cited technology as a silver lining of the pandemic, mentioning that they learned how to use tools like Zoom to build and maintain their relationships.

**Table 2:** COVID-19 silver lining identified from manual content analysis.

**Topics**	**Example**	**Participants reporting silver lining type**	**No. %**
Quality time with loved ones	“I'm in touch with my family who [lives] faraway a lot more. Kids are starting to help more in the house with cleaning and cooking. I'm getting two walks a day with my husband therefore having more quality time together!”	1,538/3,113	46.4%
Community coming together	“People reaching out to friends and family to make sure they are okay—physically and emotionally. People helping neighbors who can't go to the store on their own. People saying hello (at a safe distance) on the trails—respecting the rules but also the importance of staying friendly/being civil. People ordering from local restaurants and other ways to keep local businesses solvent.” “I love seeing the kindness and generosity of my community.”	544/3,113	16.4%
Life slowing down	“Having real time to do nothing, guilt- and FOMO-free, and the headspace to take up low-stakes hobbies just for fun, as in caring more about enjoyment than talent.” “Self introspective reflection, thinking about what really matters, getting closer to God.”	962/3,113	29%
Feeling gratitude for what they have	“Greater appreciation for family, friends, and health.” “Appreciating the small things in life that have disappeared.”	451/3,113	13.6%
Impetus for positive social change	“After this, we will be more aware of the damage we have caused, and everything will have a change. We are going to be cleaner people.” “My therapy and psychiatry firm previously refused to do appointments via telehealth. Given the pandemic, they have been forced to adopt telehealth practices. I'm hopeful that they will continue this practice afterwards.”	253/3,113	7.6%
Positive environmental impact	"Greenhouse gas emissions are down.” “Mother nature is healing as there is less pollution, less people outside and most of all ozone layer is healing.” “There is no traffic on the road, it is very quiet. This will be good for the planet.”	185/3,113	5.6%
Improved health and health literacy	“Trying to prioritize sleep and my physical and mental well-being. Doing all my cooking at home and not buying take-out/restaurant/convenience food.” “Increased awareness of hygiene.”	340/3,113	10.2%
Benefits of working from home	“I don't have to drive 1.5 h to and from work daily.” “I'm finding I am much more productive.” “I think it is good that society is finally learning how to effectively work remotely from home. Hopefully, working remotely options will remain for many workers on a full-time basis or several times per week helping many workers to have more of a work-life balance after this crisis moving forward.“	421/3,113	12.7%
Benefits of technology use	“Friends and family members are getting much better at technology and joining me on Facebook and Instagram where I've always done a lot of my socialization. So my physical contact with the world is a lot smaller but my community also feels a lot bigger and closer now.”	431/3,113	13.0%

In addition, approximately a third of participants (29%) found benefits in the “slowed down” pace of life during the pandemic. They enjoyed having more time to rest, work on professional projects, pursue new hobbies or skills, and reflect on their lives. More specifically, some (13.6%) also felt that their experiences of the pandemic urged them to be more grateful for what they had. Still others (12.7%) explicitly mentioned the benefits of being able to work from home, such as enjoying the increased flexibility of work hours and the absence of stressful commutes. Finally, a subset of participants (5.6%) viewed the reduction of human travel and activity as a silver lining because it lead to a reduced impact on the environment.

People also found silver linings in the positive effects they anticipated would happen in the wake of the pandemic. Some (10.2%) felt that the necessity of taking health precautions to prevent the spread of COVID-19 would lead to improved health literacy among the general public, and subsequently lead to improved collective health in the future. In addition, some (7.6%) felt that the severity of the pandemic would serve as an impetus for positive societal changes.

## Discussion

### Principal Findings

The goal of this paper was to identify the silver linings that individuals reported finding in the COVID-19 pandemic. During times of hardship, the act of finding silver linings, or benefits, can help people persevere by orienting them to the presence of protective factors in their lives.

We found that although people's emotions were impacted by COVID-19 and distressing events from the year, people were able to find sources of positive emotion in their lives. The public's sentiment score remained positive overall despite the tragedies and stressors that occurred in early stages of the pandemic. Using computational and qualitative natural language processing techniques, we found nine themes of silver linings. They found strength and hope in having quality time with loved ones, seeing local and global communities coming together, feeling the pace of life “slow down,” enjoying the benefits of working from home, appreciating the benefits of technology use, and viewing the pandemic as an impetus for positive societal changes, particularly improved health literacy and improvements to the environment.

### Silver Linings in the Pandemic: Denoting the Presence of Protective Factors

Although the pandemic took a severe toll on collective well-being, we found that people were able to find silver linings to their experiences. This is consistent with previous work on benefit-finding in the aftermath of traumatic events (Folkman, 2008). Positive psychology research has found that people in adverse situations often report finding different kinds of benefits from their experiences, despite its negative impact (Affleck and Tennen, 1996; Werner, 2000; Tennen and Affleck, 2002; Folkman, 2008).

The process of identifying benefits may be helpful because it helps orient people to the presence of *protective factors* in their lives, which are “skills, strengths, or resources that can help them deal more effectively with stressful events” that serve as psychological buffers that protect individuals from the potential harms of adverse situations, such as living through a pandemic [Fuller-Iglesias et al., 2008; Substance Abuse and Mental Health Services Administration (SAMHSA), 2019; Conversano et al., 2020; Magson et al., 2020; Maine Center for Disease Control Prevention, 2020; Bearden and Karlsgodt, 2021]. Reflecting on silver linings may help people better recognize the *external protective factors* in their lives—such as having strong relationships with family or friends—or their own *internal protective factors*—such as having dispositional mindfulness or practicing gratitude (Wood et al., 2008; Lomas et al., 2014; Vieselmeyer et al., 2017; Gee, 2021). As a result, they are better positioned to take advantage of these resources.

In our study, the silver linings that emerged from our analyses revolved broadly around finding a sense of community, closeness, and gratitude—all of which may help orient people to the presence of protective factors in their lives. For example, some people reported feeling a sense of increased closeness with their loved ones and found that the pandemic gave them a unique opportunity to spend quality time with their families. For these people, the act of actively recognizing and appreciating their close relationships as a “silver lining” may help them engage in meaning-focused coping with the pandemic by finding a source of positive meaning in their experiences (Folkman, 2008). In addition, this silver lining denotes the presence of the protective factor of having a supportive or valued relationship with their loved ones, which has been linked to improved psychological well-being and resilience in the face of threats (Fuller-Iglesias et al., 2008). Indeed, a recent study found that people with greater perceived family support were less lonely and had better mental health during the COVID-19 crisis while social distancing measures were in place (Li and Xu, 2020).

Similarly, the silver lining of “appreciating what one has” denotes the presence of an important internal protective factor which is the practice of gratitude. People who reported this silver lining felt an increased sense of appreciation for what they had and a greater sense of clarity about what mattered in their life. They expressed appreciation for some of the positive lifestyle changes that have emerged, reflecting on the normalization of working from home, greater awareness of physical and mental health needs, and novel uses of technology that have allowed individuals and communities to connect when in-person interactions were limited. Extensive studies have demonstrated the beneficial effects of gratitude on well-being (Sansone and Sansone, 2010; Wood et al., 2010). In the context of the pandemic, a number of studies have found that practicing gratitude and appreciation for the positive aspects of one's life protected individuals against some of the harmful mental health impacts of the pandemic among a variety of communities (Butler and Jaffe, 2021; Nguyen and Le, 2021).

Many people also found a silver lining in seeing their communities come together to support one another in a time of crisis. For some, this entailed seeing their neighbors and peers join together to help others in their local community. For others, this meant seeing people like healthcare workers, essential workers, and volunteers work on a global scale to combat the virus. Previous research on recovery from traumatic events has found that there can often be a collective outpouring of support and prosocial behavior in the aftermath of the crisis (Zaki, 2019). However, it is not always easy to recognize this, particularly in times of distress. Reporting this silver lining may denote an orientation toward the protective factors of seeing the “positive” in other humans, to focus on what can be controlled, and to draw a sense of strength from adversity at the individual and collective level (McCrae, 1984; Tedeschi and Calhoun, 2004). Because of this orientation, people who recognize this sense of communality may be better able to participate in helping others or accessing forms of support at the community level.

Notably, many individuals found solace in viewing the pandemic as an impetus for necessary positive societal changes—-a call to action to address urgent issues such as climate change, misinformation, and disparities in health and healthcare. Indeed, work on resilience through adverse life events has demonstrated that individual resilience is closely linked to interactions with systems-level factors, such as access to resources and public policy (Gee, 2021). Our results suggest that having a vision of a better future may be a key protective factor that helps individuals persevere through times of grief and uncertainty. Critically, however, belief in a better, more equitable future of a post-COVID world must be matched with concrete actions to make that vision a reality.

### Limitations

Limitations to our research includes the non-representative nature of our sample and relatively small dataset used, which may affect the NLP methods, such as word representations and the creation of a relevant vocabulary coverage of COVID-19. For example, it is possible survey respondents were able to work from home or had not lost loved ones at the time of response.

Moreover, the NLP-based topic extraction approach we implemented allowed to improve the performance when revealing the most common topics, grouping similar responses, and in translating the unstructured response to input features for the clustering algorithm. However, it is worth to mention that there is currently no standard way of combining topics with pretrained contextual representations such as BERT.

## Conclusion

By definition, “silver linings” are the signs of hope and strength, however faint, in an otherwise tragic situation. While no silver lining may be able to mitigate the impact of the pandemic on people's lives, our results show what silver linings people have turned to in order to persevere through the challenges of the pandemic. In a time of limited resources, understanding the silver linings that have given people hope, strength, and solace can inform efforts to support individual and collective recovery from the psychological and emotional challenges of the pandemic. As a result, we may be better able to heal from this crisis and better prepared to respond to potential future crises.

## Data Availability Statement

The data used in this study can be made available upon reasonable request to the corresponding author.

## Ethics Statement

The studies involving human participants were reviewed and approved by Stanford's Institutional Review Board and all participants consented to the study. The patients/participants provided their written informed consent to participate in this study.

## Author Contributions

JALV and AL conceived and designed the study, performed the analysis and interpretation of data, wrote the initial draft, and revised subsequent versions. JALV set up the natural language processing applications. AL manually annotated the survey dataset. EL, NL, and JH as senior investigators and provided relevant feedback. EL led and supervised the project. All authors read, revised, and approved the final manuscript.

## Conflict of Interest

The authors declare that the research was conducted in the absence of any commercial or financial relationships that could be construed as a potential conflict of interest.

## Publisher's Note

All claims expressed in this article are solely those of the authors and do not necessarily represent those of their affiliated organizations, or those of the publisher, the editors and the reviewers. Any product that may be evaluated in this article, or claim that may be made by its manufacturer, is not guaranteed or endorsed by the publisher.
